# First-line treatment with bortezomib rapidly stimulates both osteoblast activity and bone matrix deposition in patients with multiple myeloma, and stimulates osteoblast proliferation and differentiation *in vitro*

**DOI:** 10.1111/j.1600-0609.2010.01485.x

**Published:** 2010-10

**Authors:** Thomas Lund, Kent Søe, Niels Abildgaard, Patrick Garnero, Per T Pedersen, Tina Ormstrup, Jean-Marie Delaissé, Torben Plesner

**Affiliations:** 1 Department of Clinical Cell Biology, IRS -CSFU, University of Southern Denmark Vejle HospitalVejle, Denmark; 2 Department of Hematology, Odense University HospitalOdense, Denmark; 3 INSERM Research Unit 664Lyon, France; 4 Department of Radiology, Vejle HospitalVejle, Denmark; 5 Department of Internal Medicine and Haematology, IRS -CSFU, University of Southern Denmark, Vejle HospitalVejle, Denmark

**Keywords:** multiple myeloma, bone disease, bortezomib, osteoblast, osteolysis

## Abstract

**Objectives:**

The aim of the study was to investigate the effect of bortezomib on osteoblast proliferation and differentiation, as well as on bone matrix deposition for the first time in bisphosphonate-naïve, previously untreated patients with myeloma.

**Methods:**

Twenty newly diagnosed patients received four cycles of bortezomib treatment, initially as monotherapy and then combined with a glucocorticoid from cycle two to four. Bone remodeling markers were monitored closely during treatment. Furthermore, the effects of bortezomib and a glucocorticoid on immature and mature osteoblasts were also studied *in vitro*.

**Results:**

Treatment with bortezomib caused a significant increase in bone-specific alkaline phosphatase and pro-collagen type I N-terminal propeptide, a novel bone formation marker. The addition of a glucocorticoid resulted in a transient decrease in collagen deposition. *In vitro* bortezomib induced osteoblast proliferation and differentiation. Differentiation but not proliferation was inhibited by glucocorticoid treatment.

**Conclusions:**

Bortezomib used as first-line treatment significantly increased collagen deposition in patients with multiple myeloma and osteolytic lesions, but the addition of a glucocorticoid to the treatment transiently inhibited the positive effect of bortezomib, suggesting that bortezomib may result in better healing of osteolytic lesions when used without glucocorticoids in patients that have obtained remission with a previous therapy. The potential bone-healing properties of single-agent bortezomib are currently being explored in a clinical study in patients who have undergone high-dose therapy and autologous stem cell transplantation.

Multiple myeloma (MM) is a malignant disorder characterized by the accumulation of neoplastic plasma cells in the bone marrow. Most patients suffer from osteolytic lesions that cause pain and reduced quality of life, and may result in hypercalcemia, fractures, and spinal root or spinal cord compression. At diagnosis 80% of patients already have pathological bone findings when evaluated with conventional radiography (1).

MM causes increased bone resorption through osteoclast activation (2–6), and bone resorption is increased even in patients without osteolytic lesions (7). In addition, MM also causes decreased bone formation (8–11). Bone formation is primarily impaired in patients with overt osteolysis, whereas no decrease in bone formation is observed in MM without osteolysis or in monoclonal gammopathy of undetermined significance (MGUS) (7, 12). Likewise, osteoblasts from patients with MM with bone disease are more prone to apoptosis compared to osteoblasts from patients with MM without bone disease (13). Thus, it appears that osteolysis becomes apparent only when increased bone resorption is not compensated for by the formation of new bone (i.e. ‘imbalanced remodeling’).

Bisphosphonates, which are the only registered drugs for the treatment of osteolysis in MM (14), mainly target the increased bone resorption caused by osteoclasts (15). The very significant inhibition of osteoclasts obtained by treatment with potent bisphosphonates may in fact inhibit bone formation as some osteoclast activity seems to be important for the stimulation of osteoblasts (16). Indeed, a 50% reduction of osteoblast activity has been observed after initiation of bisphosphonate treatment (17). Accordingly, the protection against osteolysis offered by bisphosphonate treatment is only partial (18). Furthermore, osteoblast function remains impaired when MM has been brought into remission with standard chemotherapy (19), and healing of established bone lesions is rarely observed (20).

Evidence suggesting that bortezomib, the first clinically available proteasome inhibitor, could overcome the osteoblast inhibition observed in MM was provided from the APEX trial where serum alkaline phosphatase (AP) was found to be increased in patients responding to bortezomib treatment (21). It was clearly demonstrated that the stimulatory effect on bone formation was induced by bortezomib as no increase in AP was observed in patients responding to the treatment arm lacking bortezomib. Subsequent clinical studies confirmed and extended this observation, showing that the increase in AP was caused by the bone-specific alkaline phosphatase (bALP) (22–24). Cell culture studies on human as well as murine osteoblast cell lines and animal studies have demonstrated that bortezomib stimulates osteoblasts and bone formation (25–30). Furthermore, Giuliani *et al.* (31) demonstrated that bortezomib treatment increases the number of osteoblasts in the bone marrow of patients with MM. So far, however, the clinical studies have been performed in patients with relapsing MM who have received prior chemotherapy, treatment with bisphosphonates, and frequently have also been treated with glucocorticoids, which are known to inhibit bone formation (32, 33).

In this study, we report that bortezomib used alone and in combination with a glucocorticoid as front-line treatment in previously untreated, bisphosphonate-naïve patients stimulates bone formation not only as shown by an increase in bALP but also through the use of a novel bone marker, pro-collagen type I N-terminal propeptide (PINP) that is released as collagen is deposited and becomes insoluble during the formation of the organic bone matrix (34) and may, therefore, be a better marker for ongoing bone formation. We also found that the formation of new bone, as reflected by an increase in serum PINP, was inhibited by the addition of a glucocorticoid to the treatment with bortezomib and extended the observations by *in vitro* studies demonstrating that pulse-treatment with bortezomib induced osteoblast differentiation which was inhibited by glucocorticoid treatment, while osteoblast proliferation was not affected.

## Material and methods

The study was conducted as a prospective bicenter non-randomized phase II clinical trial in accordance with the Helsinki declaration and ‘Good Clinical Practice’ guidelines, and was approved by the Danish Medicines Agency, the Regional Ethical Committee, and the Danish Data Protection Agency. All patients gave informed consent. The study received EudraCT number 2006-002464-26 and trial registration number NCT00436059 at clinicaltrial.gov.

Only previously untreated newly diagnosed patients with MM were included in the trial. Patients who had previously received bisphosphonate treatment intravenously or orally for any indication were excluded from the study. A performance status of three or better and a life expectancy of at least 3 months and measurable disease were required for inclusion. All patients received four cycles of bortezomib 1.3 mg/m^2^. Each cycle lasted 3 wk and bortezomib was administered by i.v. push on day 1, 4, 8 and 11. The first cycle was given as monotherapy and in the three subsequent cycles bortezomib was given in combination with dexamethasone 20 mg on the day of bortezomib infusion and on the following day.

### Patients

Twenty consecutive patients from Vejle Hospital or Odense University Hospital were included in the trial (ten males and ten females). The median age of the patients was 68.5 yr (range: 51–85 yr), 15 patients had IgG myeloma, three patients IgA myeloma and two patients had light chain disease only. Response was monitored at least every third week, prior to the initiation of a new treatment cycle by measurements of serum M-component (IgG or IgA), serum-free light kappa/lambda chains (FLC), creatinine, ionized serum calcium and other relevant laboratory parameters. Likewise, a careful physical examination, evaluation of performance status, registration of side effects and concurrent medication were conducted every 3 wk. Thrombocyte levels were measured prior to each bortezomib infusion.

After four treatment cycles, treatment response was evaluated according to the International Uniform Response Criteria for Multiple Myeloma (35).

### Markers of bone turnover

The bone turnover markers bALP, PINP, Dickkopf 1 (DKK-1) and N-terminal crosslinked telopeptide of type I collagen (NTX-I) were measured together with parathyroid hormone (PTH), calcium and albumin during the study period. Blood samples, used for the measurement of bone turnover, were collected in the morning from fasting patients. Urine samples were collected as fasting second void morning urine. The samples were immediately centrifuged and stored at −80°C. Samples were collected before the first infusion of bortezomib on day 1 and subsequently on days: 2, 3, 4, 8, 9, 10, 11, 21, 24, 28, 31, 42, 52, 63 and 73 of the study period. All samples from the individual patients were analyzed in the same batch to minimize analytical variation. Serum bALP was measured with a non-competitive enzyme immunoassay technique (Quidel Corporation, San Diego, CA, USA). Serum PINP was measured by a two-site immunoassay detecting both mono and trimetric forms of intact PINP (Roche Diagnostics, Hvidovre, Denmark). DKK-1, an osteoblast inhibitor, was measured in plasma using an enzyme-linked immunoassay (Biomedica, Wien, Austria). To evaluate bone resorption, we measured urine NTX-I (Ostex International, Seattle, WA, USA) by competitive enzyme immunoassay techniques. bALP, PINP, DKK-1 and NTX-I were all analyzed in duplicates. PTH was measured by an electrochemiluminescence technique that measures intact PTH (Roche Diagnostics). Calcium and albumin were measured by colorimetric methods (Roche Diagnostics).

### *In vitro* studies of osteoblasts

Human adipose-derived stem cells (ADSCs) (Invitrogen, Taastrup, Denmark) capable of differentiating into osteoblasts, and which serve as a model for pre-osteoblasts, were used to analyze the effects of bortezomib and glucocorticoid administration on osteoblastic precursor cells (undifferentiated ADSCs) and on more mature osteoblasts (differentiated ADSCs) (36–38). The cells were grown in a humidified incubator with 5% CO_2_ at 37°C. For experiments on osteoblastic precursors, the cells were grown in MesenPro RS Medium (Invitrogen) and cultured for a maximum of four passages. For experiments on more mature osteoblasts the ADSCs were taken into culture, grown for one passage and reseeded at a density of 5000 cells/cm^2^ in well plates. The cells were then cultured for 24 h in MesenPro RS Medium where after the medium was exchanged with medium from the StemPro Osteogenesis Differentiation kit (Invitrogen), and the cells were allowed to differentiate for 10 d with medium renewal every 3–4 d.

To test the effects of bortezomib and glucocorticoid treatment on undifferentiated or differentiated ADSCs, the cells were exposed during *in vitro* culture conditions to: (i) a 3-h pulse treatment with bortezomib 25 nm as described previously (39) and then placed in control medium, (ii) continuous exposure to 5 μm prednisolone (corresponding to a daily oral dose of approximately 20 mg dexamethasone) for 48 h or (iii) control medium. For all conditions, 0.5% DMSO (solvent of prednisolone) was added. The cells were harvested after 48 h for response evaluation. We chose to use pulse-treatment with bortezomib to better mimic the *in vivo* conditions where the drug is given as a bolus infusion and the serum level quickly reaches a peak after which it is rapidly taken up by the tissue and is subsequently followed by a slow release and elimination (39, 40).

### AP activity of cultured cells

Human ADSCs were washed after defined periods in culture, lysed in reaction buffer (60 mm Na_2_CO_3_, 40 mm NaHCO_3_, 0.1% TritonX-100, 2 mm MgSO_4_, 4 mm 4-nitrophenyl phosphate) and incubated in the dark for 30–60 min until a yellow color was visible. A sample of the lysate was taken aside and used to determine the protein concentration according to the instructions by the supplier of the kit (Bio-Rad Protein Assay; Bio-Rad, Copenhagen, Denmark). The remaining solution was mixed with 1 volume of 1 m NaOH and OD_405_ was measured on a plate reader (Synergy HT; Bio-Tek, Winooski, VT, USA) along with a standard curve of 4-nitrophenol. Results are given both as total AP activity and as AP activity per μg of protein in the extract. The measurements were done in five replicates.

### Cell proliferation

Undifferentiated ADSCs were seeded in culture wells at a density of 7.1 × 10^3^ cells/cm^2^ and incubated for 24 h. Subsequently, cells were pulse-treated with bortezomib as described, washed and cultured for 48 h or alternatively exposed to prednisolone for 48 h. The cells were then fixed and stained with May-Grünwald as previously described (41). Cell proliferation was determined by counting the surface in the culture well covered by cells using a graticule with 100 points inserted into the ocular using a 10× objective (Zeiss AxioVert; Carl Zeiss, Birkerød, Denmark). Wells of a 96 well-plate were divided into 19 squares using the graticule and counted. The percent cell surface area was determined as an average for the 19 squares in five replicates.

### Analysis of gene expression

For gene expression analysis, ADSCs were seeded at a density of 4.3 × 10^4^ cells/cm^2^ and incubated for 24 h using either immature or differentiated osteoblasts derived as described earlier. The cells were then either pulse-treated with bortezomib or exposed to prednisolone for 24 h as described. Medium was removed, the cell layer was washed three times in PBS followed by lysis of the cells in the plate and isolation of RNA according to the instructions by the supplier of the kit (Trizol Plus RNA Purification kit; Invitrogen). cDNA was generated using 500 ng RNA and the iScript kit (Bio-Rad) according to the instructions by the supplier. Subsequently, Q-PCR was performed using the TaqMan approach according to instructions by the supplier. The Q-PCR was run on a Realtime PCR machine (7900HT; Applied Biosystems, Foster City, CA, USA). As reference genes, Abl and GUS were used. The expression levels of both genes were converted into relative values by the use of an internal standard curve. Five replicates were done per sample. The overall average expression levels of Abl and GUS were used to normalize the expression levels of the genes of interest. All TaqMan primer/probe sets were inventoried and used according to the instructions by the supplier (Applied Biosystems) (used primer/probes: GUS, Hs99999908_m1; Abl, Hs00245443_m1; osteopontin, Hs00959010_m1; collagen type I, Hs00164004_m1).

### Statistical analysis

Changes in serum or urine levels of bALP, PINP, DKK-1, NTX-I, PTH and calcium were analyzed by comparison of the value of each marker at a given time point with the same marker pre-treatment using a Wilcoxon signed rank test. Spearman’s test was used to analyze for correlation between calcium and PTH. Urine NTX-I was normalized to urine creatinine and expressed as relative values. Calcium levels were adjusted for variations of serum albumin. Patients who left the protocol before response evaluation was possible were analyzed as responders. Cell culture experiments were analyzed using an unpaired *t*-test. When analyzing Q-PCR levels, the control values were normalized to one. All *P*-values are two sided, and the significance level was set at *P* ≤ 0.05.

## Results

Twenty patients were included in the protocol. Fourteen responded to treatment with a partial response or better, and ten of these with a very good partial response or better. Four patients responded with less than a partial response. Two patients died before the final response evaluation could be conducted, one because of cerebral hemorrhage the other because of gastro-intestinal bleeding. These two patients were evaluated as responders. Two patients underwent major orthopedic surgery preceding protocol inclusion causing abnormal high bone formation markers (42). These two patients were excluded from further analysis. Thus, fourteen patients were evaluated as responders and four patients as non-responders.

### Markers of bone turnover in patients

**Figure 1:**
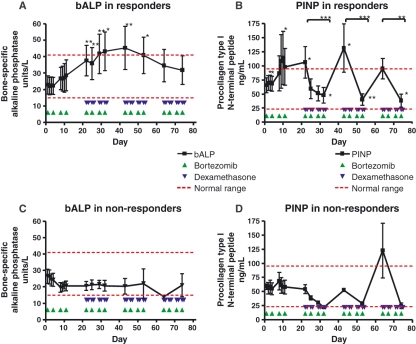
Effect of treatment according to protocol on bone-specific alkaline phosphatase (bALP) and pro-collagen Type I N-terminal peptide (PINP) in responders and non-responders. bALP increased twofold in responding patients (A). PINP showed a similar increase (B). Both values reached a maximum value at day 42. Furthermore, PINP showed a transient significant decrease every time dexamethasone was added. No significant changes were observed in non-responding patients (C–D). Response is defined as partial response or better. Results are shown as mean values ± SEM from 14 (A–B) and 4 (C–D) patients. ***P < 0.001; **P < 0.01; *P < 0.05 using a Wilcoxon signed rank test.

Both bALP and PINP increased in patients responding to bortezomib treatment (Fig. 1A,B). Their development showed many similarities. Both markers reached a peak value on day 42 after which the increase diminished. The magnitude of changes was also strikingly similar when analyzing values prior to each treatment cycle. At the beginning of cycle two, bALP and PINP had increased by 64.4% and 62%, respectively; at cycle three by 98% and 101%; and at cycle four by 51% and 45%. However, there were obvious dissimilarities when comparing bALP and PINP in responding patients. While bALP showed no fluctuation, a rapid and transient decrease of 61% on average in PINP was observed during cycle two to four. No decrease was seen in the first treatment cycle which was also the only cycle during which bortezomib was given without the addition of dexamethasone.

In patients who did not respond to treatment with a partial response or better, no significant changes in bALP or PINP were observed (Fig. 1C,D).

**Figure 2:**
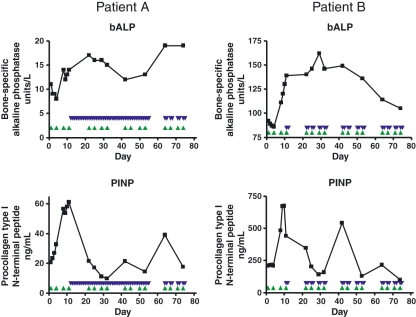
Effect on bone-specific alkaline phosphatase (bALP) and pro-collagen Type I N-terminal peptide (PINP) of altering the time point of administration of dexamethasone to two patients. Two patients received treatment with dexamethasone at day 12 and 11 respectively in a violation of the protocol. In both cases, bALP remained unchanged, whereas an immediate decline in PINP was observed.

Two patients received off-protocol treatment with glucocorticoid prior to the planned time point. Patient A received 100 mg prednisolone from day 12 to 53 because of the risk of spinal cord compression. Patient B received 20 mg dexamethasone on day 11 and 12. Both patients responded with an instant decrease in PINP, whereas bALP remained unchanged (Fig. 2).

**Figure 3:**
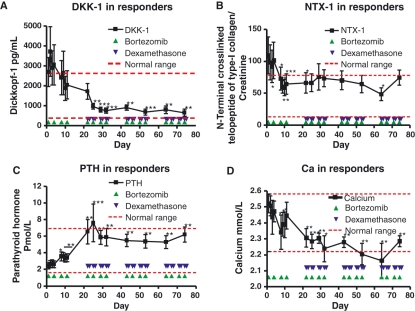
Effect of treatment on Dickkopf-1 (DKK1), N-terminal crosslinked telopeptide of type I collagen (NTX-I), parathyroid hormone (PTH), and calcium in responders. DKK-1 showed a fourfold decline in responding patients (A). No changes were observed in non-responding patients (data not shown). NTX-I declined twofold, however significance is lost after the addition of dexamethasone (B). PTH increased significantly from day 8 and onward (C). The changes in PTH were mirrored by a simultaneous decrease in calcium (D). The correlation coefficient between calcium and PTH is −0.465 *P* < 0.0001 (data not shown). Calcium levels were corrected to albumin levels. Response is defined as partial response or better. Results are shown as mean values ± SEM from 14 patients. ***P < 0.001; **P < 0.01; *P < 0.05; using a Wilcoxon signed rank test.

In responding patients, the osteoblast inhibitor DKK-1 decreased rapidly after one treatment cycle and reached a plateau at about 25% of the initial value around day 30 (Fig. 3A). In patients not responding to treatment, DKK-1 remained elevated (data not shown).

The levels of the bone resorption marker NTX-I were reduced by approximately 50% after one treatment cycle in responding patients and remained relatively constant thereafter (Fig. 3B). Patients with less than a partial response showed a small but significant decrease in NTX-I (data not shown). PTH increased significantly after only 1 wk of treatment, reaching a maximum of threefold of the baseline value at the beginning of cycle two and then leveling off into a plateau around 2.5-fold of the baseline level (Fig. 3C). The increase in PTH was inversely correlated to decreasing calcium levels, *R* = −0.465, *P* < 0.0001 (Fig. 3D). A similar but statistically insignificant trend for both calcium and PTH was found in non-responding patients (data not shown).

### Effect of bortezomib and glucocorticoid on osteoblast precursors and differentiated osteoblasts *in vitro*

**Figure 4:**
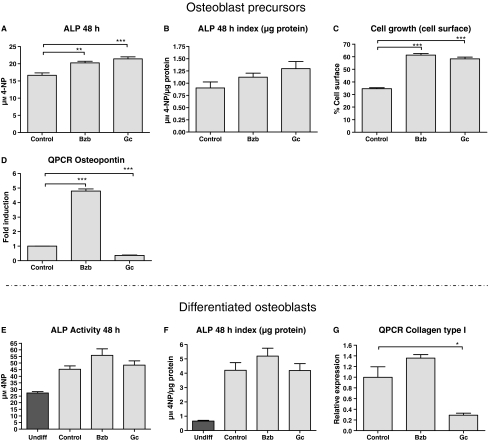
Effect of bortezomib (Bzb) and prednisolone (Gc) on osteoblast precursors and differentiated osteoblasts. In pre-osteoblasts, both treatments resulted in increased levels of alkaline phosphatase (AP) (A). The significance of this was lost when adjusting for cell proliferation (i.e. total protein, B–C). Treatment with Bzb or Gc had a substantial effect on osteoblast proliferation almost doubling the area covered by cells after only 2 d (C). Osteopontin, a maker reflecting differentiation, showed a significant fivefold increase when cells were treated with Bzb; Gc on the contrary caused a decrease in osteopontin (D). No effect was observed on AP when differentiated osteoblasts were exposed to either Bzb or Gc (E–F). Treatment of differentiated osteoblasts with Gc, however, caused a fivefold decrease in collagen type I RNA, whereas collagen type I RNA remained unchanged after bortezomib exposure (G). Cells were cultured for 48 (A–C, E–F) or 24 h (D, G). Data from undifferentiated human adipose-derived stem cells (undifferentiated) are shown in E–F. Results are shown as mean values ± SEM of either five (A–C, E–F) or three replicates (D, G). ***P < 0.001; **P < 0.01; *P < 0.05; using an unpaired *t*-test.

Human osteoblast precursors and differentiated osteoblasts were exposed to either bortezomib or prednisolone as described in the Materials and Methods section. Osteoblast precursors responded to the treatment with a significant increase in levels of AP activity (Fig. 4A), but the difference became insignificant when AP levels were adjusted to the total protein content of the lysates, suggesting that the increase in AP activity may reflect cell proliferation (Fig. 4B). This assumption was supported by the findings presented in Fig. 4C showing increased cell growth with both treatments.

The impact of bortezomib or glucocorticoid on the differentiation of osteoblast precursors was studied by measuring an early osteoblast differentiation marker, osteopontin, by Q-PCR (43). Treatment with bortezomib resulted in a fivefold increase in levels of osteopontin while treatment with the glucocorticoid caused a strong reduction of osteopontin (Fig. 4D). Thus, bortezomib seems to induce both proliferation and differentiation of osteoblast precursors, whereas the stimulation of osteoblast proliferation induced by glucocorticoides, from a bone formation perspective, was counteracted by inhibition of differentiation.

When ADSCs were induced to differentiate into more mature osteoblast-like cells before treatment with bortezomib or glucocorticoid, AP levels were not significantly different from controls (Fig. 4E,F). However, in more mature osteoblast-like cells, we found that treatment with glucocorticoid induced a less mature phenotype as shown by suppression of the differentiation marker collagen type I (Fig. 4G). Thus, our *in vitro* findings show that bortezomib and glucocorticoids may both induce proliferation of osteoblasts, and that bortezomib may also induce differentiation of osteoblast precursors while glucocorticoids appear to inhibit the differentiation of osteoblast precursors and induce an immature phenotype in mature osteoblasts.

Overall, our *in vitro* findings seem consistent with the interpretation that the increased serum bALP level in patients could be because of enhanced growth and differentiation of osteoblast precursors. The observed transient decrease in PINP probably reflects reduced collagen type I synthesis caused by treatment with the glucocorticoid.

## Discussion

Osteolysis remains a major problem in MM and approximately 80% of patients have a pathological bone status already at diagnosis. Impaired bone formation is a major contributor to the development of clinically apparent osteolysis. Over recent years, it has been shown that bortezomib, the first clinically approved proteasome inhibitor, may increase the markers of bone formation, bALP and osteocalcin, which are usually suppressed in MM. However, previously reported studies have been conducted on a very heterogeneous population of patients with MM, both with regard to earlier treatment regimens and with regard to concurrent treatment with drugs known to have an impact on bone remodeling, e.g. dexamethasone and bisphosphonates. Here, we have studied for the first time the effect of bortezomib on bone formation markers when used as frontline treatment in bisphosphonate-naïve patients. We demonstrate that treatment with bortezomib not only results in increased osteoblast activity but also in an increase in actual bone matrix deposition evaluated through the novel marker PINP (44). Owing to multiple measuring points during the treatment period, we were able to demonstrate a very early response after only 1 wk of treatment, and after the first treatment cycle both bALP and PINP levels exceeded the upper normal range of healthy adults.

The positive effect of bortezomib on bone formation as demonstrated by biochemical markers reached a maximum after 6 wks, after which a slight decline was observed. This is in perfect agreement with observations from the APEX trial, where bortezomib was given as single agent (21). One explanation for the biphasic development of the curve could be that an initial direct osteoblast stimulation caused by bortezomib is later counteracted by a decrease in total bone remodeling as indicated by a decrease in the bone resorption marker NTX-I. The decline observed in NTX-I was probably caused partly by a reduced tumor burden, partly because of a direct inhibitory effect of bortezomib on bone resorption by osteoclasts (39, 45).

The delay of the addition of glucocorticoid treatment in this study until cycle two made it possible to discriminate to some extent between the effect of bortezomib as single agent vs. the effect of the combination of bortezomib and dexamethasone. Unlike bALP which was relatively unaffected by the addition of the glucocorticoid, PINP showed a profound but transient decline each time dexamethasone was given to the patient. As PINP better reflects actual bone deposition than bALP, our finding suggests that the beneficial effect of bortezomib on bone formation *in vivo* is partly inhibited by co-treatment with a glucocorticoid. To elaborate further on this, we conducted *in vitro* experiments on both osteoblast precursors and more differentiated osteoblasts.

It has previously been shown in cell culture studies on human as well as murine osteoblast cell lines and in animal models that bortezomib may induce proliferation and differentiation of osteoblast precursors and stimulate bone formation (25–30). In our *in vitro* model of human osteoblast precursors, we found that not only bortezomib but also glucocorticoid induced proliferation of osteoblast precursors (46). However, unlike bortezomib, which stimulated osteoblast differentiation, treatment with glucocorticoid inhibited differentiation (47, 48). The inhibition of osteoblast maturation by the glucocorticoid was found both in osteoblast precursors (reduced osteopontin) and in more mature osteoblasts (reduced collagen type I). The expression of osteopontin by osteoblast precursors and collagen type-1 by more mature osteoblasts was suppressed by the glucocorticoid in both cases to levels that were lower than the untreated control, suggesting that glucocorticoids not only inhibit differentiation, but may actually induce de-differentiation of osteoblast precursors and osteoblasts. This is probably reflected in the clinical study by a significant drop in PINP levels, indicating the loss of osteoblast maturity and capacity to form the organic bone matrix by deposition of collagen type I. The persistently elevated serum bALP in the clinical study could suggest that bALP is clinically less relevant as a marker than PINP, as bALP may just reflect proliferation of osteoblast precursors with or without the capacity to form bone.

In the literature, the increased osteoblast activity seen after bortezomib treatment has been ascribed to a decrease in the osteoblast inhibitor DKK-1. We observed a rapid decrease in DKK-1 after one treatment cycle in responding patients. However, it has been shown that DKK-1 also declines in responding patients receiving non-bortezomib-containing regimes (49). This result corresponds well with DDK-1 over-expression being induced by the myeloma cells (11). However, only bor-tezomib-containing regimens have resulted in increased osteoblast activity. In our study, there was no increase in bALP in patients without response to treatment. Non-responders also had elevated or stable DKK-1 levels. A possible explanation as to why we observed no increase in bone formation markers in non-responding patients, despite a direct positive effect of bortezomib on osteoblasts precursors, could be that a continued high myeloma tumor burden resulted in high levels of osteoblast inhibitors e.g. DKK-1 abrogating any such effect. This hypothesis is in accordance with an observation showing that patients with lower levels of DKK-1 have greater increases of bALP (24).

In the paper by Zangari *et al.* (21), an increase in PTH was noted along with an increase in bALP. In our study, we observed a similar increase in PTH, which was tightly correlated to, and may have been caused by a simultaneous decrease in calcium levels. Indeed, patients went from having calcium levels in the upper normal range to outright hypocalcaemia. Treatment with oral calcium during this period could perhaps improve healing of osteolytic bone lesions.

Here, we show that bortezomib when used in previously untreated and bisphosphonate-naïve patients increases both osteoblast activity and bone matrix deposition. When using computer tomography, we could in two cases demonstrate signs of actual healing only 3 months after initiation of therapy. The positive effect of bortezomib is probably mediated through increased proliferation and differentiation of osteoblast precursor cells. However, we also show that the addition of a glucocorticoid to bortezomib may inhibit the beneficial effect of bortezomib on bone formation. The inhibition of osteoblasts by glucocorticoids was not detected by the commonly used marker of bone formation, bALP. Regarding MM treatment strategies, our results may suggest that while at the time of diagnosis of MM the combination of bortezomib and glucocorticoids with or without the addition of other drugs is needed to obtain rapid control of the disease, later in the course of the disease, when myeloma as such is well under control, single-agent treatment with bortezomib may be justified in order to improve healing of osteolytic lesions. A clinical study in which patients with MM are randomized to treatment with bortezomib or no treatment after high-dose therapy and autologous stem cell transplantation with a focus on bone healing has now been initiated (EudraCT:2008-004264-39).
